# Exploring optimal microscopic keyhole access to the skull base: an anatomical evaluation of transciliary supraorbital and transpalpebral orbitofrontal craniotomy approaches

**DOI:** 10.1007/s10143-024-02554-2

**Published:** 2024-07-16

**Authors:** Romel Corecha Santos, Bhavika Gupta, Mohammadmahdi Sabahi, Rocco Dabecco, Raphael Bastianon Santiago, Edinson Najera, Brandon Kaye, Badih Adada, Alexander Yu, Mauricio Mandel, Hamid Borghei-Razavi

**Affiliations:** 1 Department of Neurological Surgery, Pauline Braathen Neurological Center, Cleveland Clinic Florida, Weston, FL USA; 2 Department of Neurosurgery, Allegheny General Hospital, Pittsburgh, PA USA; 3 Dr. Kiran C. Patel College of Allopathic Medicine, Nova Southeastern University, Fort Lauderdale, FL USA

**Keywords:** Transciliary, Transpalpebral, Keyhole, Skull base

## Abstract

Purpose: The past two decades have witnessed the rise of keyhole microscopic minimally invasive surgeries, including the transciliary supraorbital approach (TCA) and transpalpebral approach (TPA), commonly known as the transorbital approach. This study aims to elucidate the nuances, specific indications, and advantages of each approach. Methods: A series of dissections were conducted on five formalin-fixed, alcohol-preserved cadaver heads. The TCA was performed on one side, and the TPA on the other. Virtual measurements of working angles for both approaches were recorded. Additionally, three clinical cases were presented to illustrate the practical application of the techniques. Results: For TCA, the craniotomy dimensions were 1.7 cm x 2.5 cm (Cranial-Caudal (CC) x Lateral-Lateral (LL)), while for TPA, they measured 2.1 cm x 2.9 cm (CC x LL). The measurements of anterior clinoid processes (ACP) were obtained and compared between approaches. In the TCA, the mean ipsilateral ACP measurement was 62 mm (Range: 61 –63 mm), and the mean contralateral ACP measurement was 71.2 mm (Range: 70 –72 mm). In TPA, these measurements were 47.8 mm (Range: 47 –49 mm) and 62.8 mm (Range: 62 –64 mm), respectively. TCA exhibited an average cranial-caudal angle of 14.9°, while TPA demonstrated an average of 8.3°. Conclusion: The anterior cranial fossa was better exposed by a TCA, which also featured shorter operative times, enhanced midline visualization, and a quicker learning curve. Conversely, the middle fossa was better exposed by a TPA, making it an excellent option for middle fossa pathologies, including those in the anterior temporal lobe. After sphenoid bone wing drilling, the TPA offers superior visualization from the lateral to the medial aspect and enhances the CC angle. Additionally, the TPA reduces the risk of postoperative frontalis palsy based on anatomic landmarks. However, the TPA requires a greater cranial osteotomy, and due to unfamiliarity with eyelid anatomy, the learning curve for most neurosurgeons is lengthier for this procedure.

**Supplementary Information:**

The online version contains supplementary material available at 10.1007/s10143-024-02554-2.

## Introduction

A minimally invasive alternative to the frontotemporal (pterional) craniotomy was first described as the keyhole supraorbital approach. Since its initial description, the approach was further modified and popularized to treat a variety of vascular pathologies [1, 2]. What is now ubiquitously referred to as the “eyebrow craniotomy”, the transciliary approach (TCA) is commonly used to treat a myriad of skull base pathologies [1, 3]. The supraorbital, eyebrow approach offers the distinct advantage of quick exposure, a small surgical footprint with excellent visualization and maneuverability, and better cosmesis. It minimizes significant injury to the surrounding soft tissues such as the supraorbital neurovascular bundle, facial nerve, and other frontotemporal structures [4].

Conversely, the transpalpebral transorbital approach (TPA) is a relatively novel minimally invasive route to access the anterior and middle cranial fossa [5]. Additionally, unlike the TCA corridor, the TPA has excellent access to the middle fossa, distal Sylvian fissure and temporal lobe region [6]. Selection of the optimum surgical approach relies on its overall ability to create satisfactory exposure of the lesion while minimizing collateral damage to adjacent soft tissue and neurovascular structures [7, 8]. In addition, cosmetic results cannot be neglected. It has often been debated that transciliary and transpalpebral approaches are similar in exposure, and the use of one over the other is biased based on the preference of the surgeon or the institution’s culture. While there is ample literature discussing the indication of each approach for specific pathologies [9], there are no anatomical studies comparing them with a focus on the anterior and middle cranial fossa, the neuroanatomic landmarks and the advantages and disadvantages of each approach.

The aim of our paper is to compare the transciliary and transpalpebral approaches in microscopic surgeries from an anatomical point of view. We discuss the extent of access provided by the two approaches and support our discussion with the use of illustrative cases.

## Materials and methods

### Cadaveric preparation

Both approaches were performed at Cleveland Clinic Florida Skull Base Microsurgery Laboratory on five formalin-fixed, alcohol-preserved cadaver heads injected with colored silicone. The red dye was used for the arteries and the blue dye for the veins. All dissections were performed bilaterally using standard microsurgical instrumentation with 6.4x to 40x magnifications (M320 Leica^®^). The transciliary supraorbital approach was completed on one side, and the transpalpebral transorbital approach was completed on the other side. The specimens were placed in a surgical position using a table-mounted Mayfield clamp. Each dissection step was photographed with a camera (Nikon^®^ SS-MS1).

### Positioning

The heads were placed supine and held by a three-pin skull fixation device (Mayfield^®^ clamp model). Two ipsilateral pins were fixed at the mastoid region, while the contralateral pin was secured at the superior temporal line above the temporal muscle. The heads were kept elevated at 30° and extended at 20° while keeping the malar eminence as the superior landmark. The heads were rotated 15^o^-30^o^ toward the contralateral side to provide an unobstructed view of the anterior cranial fossa.

### Assessment of working angles and surgical trajectories

A non-contrast head computed tomography (CT) scan was obtained for all five specimens before the anatomical dissections. The working angles for the transciliary and transpalpebral approaches were measured virtually using 2 and 3-dimensional (3D)-reconstructed images with the anterior clinoid process (ACP) and skull base as landmarks (Weiss DICOM viewer V4.0.3 FLOSS). The distances between the craniotomy and the intracranial landmarks (ipsilateral ACP, contralateral ACP, interpeduncular cistern, posterior clinoid process, sphenoid wings, internal carotid artery (ICA) bifurcation, and Middle cerebral artery (MCA) bifurcation) were obtained through a neuronavigation system (Brainlab AG, Munich, Germany) and were based on the CT scan of all specimens.

### Dissection technique

#### Transciliary approach

**Fig. 1:**
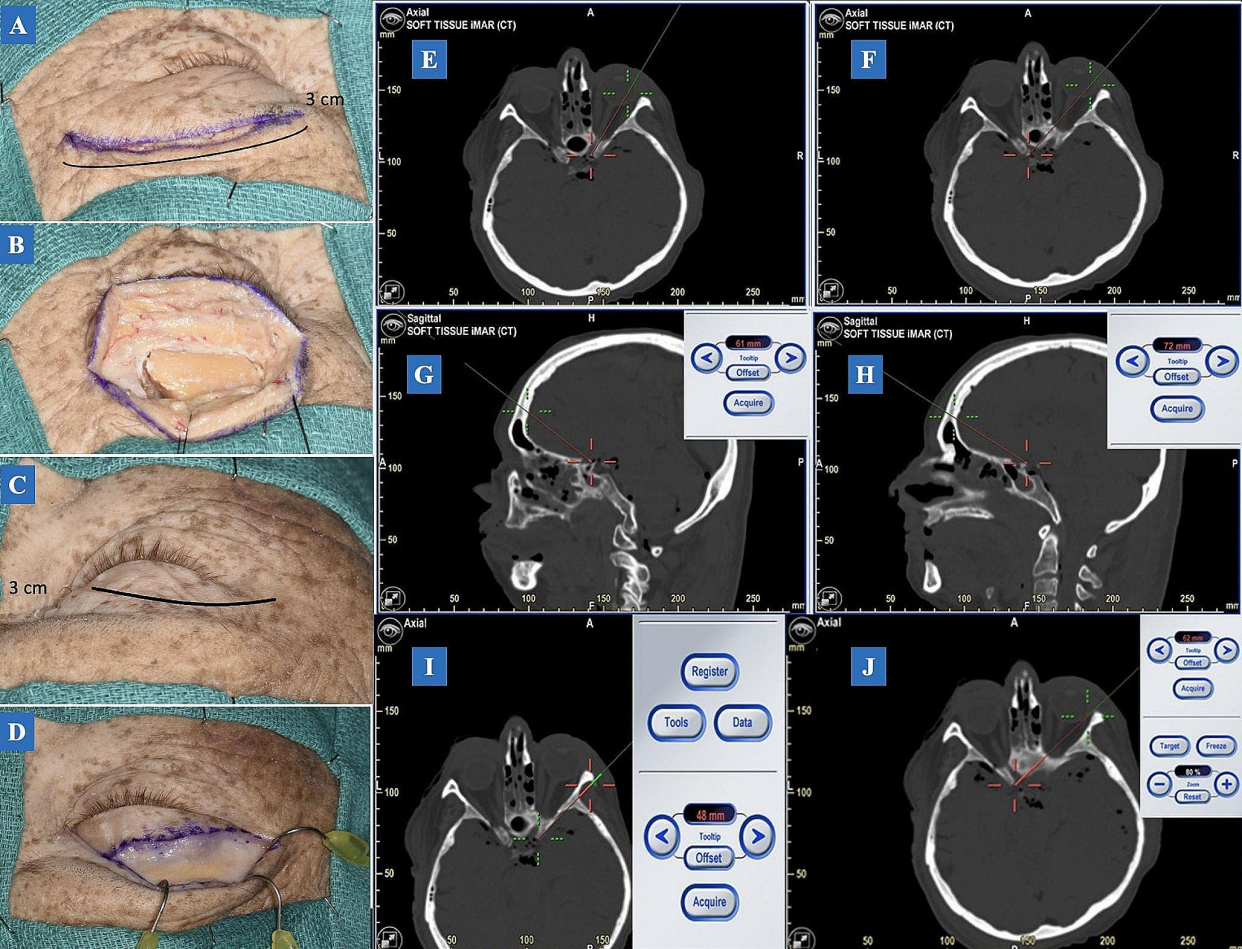
**A**: An incision with a 15-scalpel blade was performed on the left eyebrow. The incision length is approximately 3 cm with an extension of 5 mm laterally for access to the MacCarty keyhole. The supraorbital notch containing the neurovascular complex must be preserved medially. **B**: The craniotomy was exposed with a dimension of 1.7 cm x 2.5 cm (CC x LL). **C**: A 3.0 cm incision with a 15-scalpel blade was performed on the right upper eyelid crease. **D**: A lateral extension of 1.5 cm will allow access along the temporal region. This extension must be above the lateral canthal tendon. **E–H**: Ipsilateral and contralateral anterior clinoid process measurement in the TCA. Considering the five specimens, the TCA mean was 62 mm for the ipsilateral anterior clinoid process and 71.2 mm for the contralateral anterior clinoid process. The measurement was from the inner and upper edge of the craniotomy. In addition, analyzing the angle of attack and having the ipsilateral anterior clinoid process as the landmark, the TCA allowed a cranial-caudal angle average of 14.4◦, whereas the TPA was 8.1◦. **I**, **J**: Ipsilateral and contralateral anterior clinoid process measurement in the TPA. Considering the five specimens, the TPA mean was 47.8 mm for the ipsilateral anterior clinoid process and 62.8 mm for the contralateral anterior clinoid process. The measurement was from the craniotomy inner edge. *Abbreviations*: TCA: transciliary approach; TPA: transpalpebral approach; CC: cranial-caudal; LL: lateral-lateral

A three-centimeter incision was made within the eyebrow with a 15-scalpel blade and extended beyond the eyebrow by half a centimeter to allow for access to the MacCarty keyhole. The supraorbital notch containing the neurovascular complex was preserved medially. The frontalis muscle was incised along with the skin. Then, the periosteum was dissected and reflected inferiorly in a C-shape fashion, thus exposing the frontal bone to the frontosphenoidal suture laterally. The temporal muscle was detached from the superior temporal line. Once the bone was exposed, one burr hole was drilled 5.0 mm superior from the pterion at the MacCarty keyhole to expose the anterior frontal and temporal dura mater. A bone flap was created using a craniotome drill with a footplate attachment. The incision extension has represented the main determinant for the craniotomy size. The orbital rim was not included in the bone flap. This decision was based on the clinical setting, as most neurosurgeons worldwide perform a transciliary approach with no orbital rim osteotomy [10]. The posterior table of the orbital rim was drilled flush **(**Fig. 1A and B**)**.

Next, the dura mater was opened in a C fashion based on the orbital rim. The dissection continued till the anterior cranial fossa was exposed. Both ACPs were identified and measured from the internal edge of the craniotomy. The ipsilateral optic nerve, internal carotid artery, optic chiasm, and contralateral optic nerve were dissected. Next, an intradural partial anterior clinoidectomy was completed to facilitate partial dissection of the Sylvian fissure. Finally, the anterior communicating complex was identified and dissected, followed by the opening of the lamina terminalis. Thus, allowing access to the anterior portion of the third ventricle. Despite the deep surgical corridor, the interpeduncular cistern was accessible.

#### Transpalpebral approach

A 3.0 cm incision was made on the upper eyelid crease with a 15-scalpel blade. The incision extended from the supraorbital notch to the temporal region. Care was taken to not violate the orbicularis muscle. The incision was above the lateral canthal tendon, extending 1.5 cm along the temporal region (Fig. 1C and D). The orbicularis muscle was dissected and opened horizontally, exposing the orbital septum. The orbital rim was palpated, and a sub-periosteal dissection was completed exposing the neurovascular bundle medially and the frontal process of the zygoma laterally. The neurovascular complex within the supraorbital notch was preserved. The dissection continued into the orbital compartment, separating the periorbita from the bone. The components of the orbital roof and lateral wall were identified. Subsequently, the frontal periosteum was dissected vertically and horizontally, preserving the supratrochlear nerve medially and extending below the frontozygomatic suture. Ipsilateral and contralateral ACP measurement in the TCA and TPA showed in Fig. 1E, F, G, H and J.

**Fig. 2:**
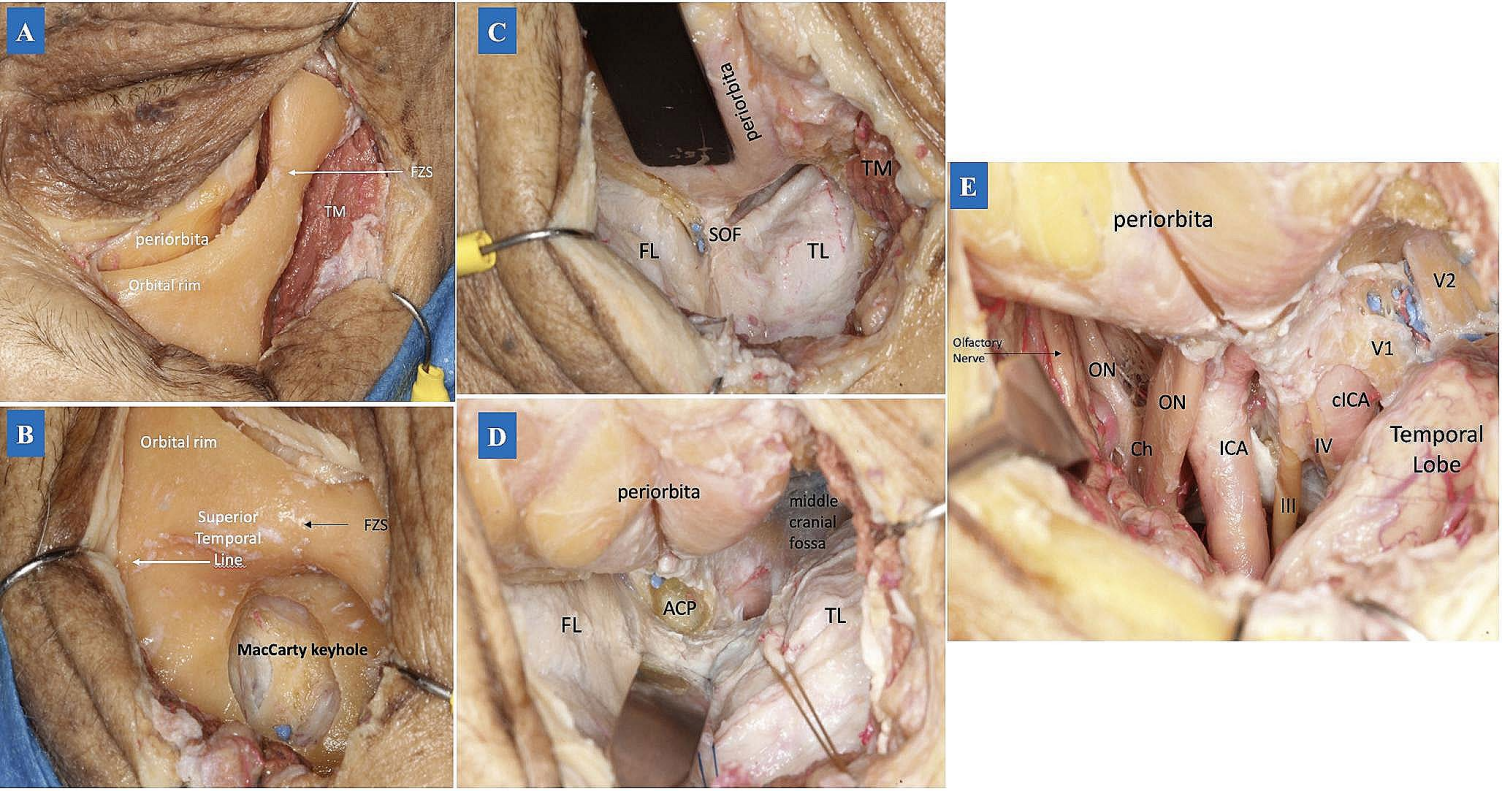
**(A)** After the temporal muscle was detached from the superior temporal line, the periorbita was also detached from the orbital bone. Then the periosteum was dissected laterally, exposing the frontozygomatic suture. This step is essential to access the temporal lobe and middle cranial fossa. **(B)** A large burr hole at the MacCarty keyhole using the craniotome drill with a footplate attachment was performed. Then the frontoorbital bone was excised in one-piece, exposing the frontal and temporal dura mater and periorbita. **C**: After drilling the orbital roof and the sphenoid wing, it was possible to access the superior orbital fissure and a wide frontal and temporal lobe area. **D**: A peeling of the middle cranial fossa was performed beyond an extradural anterior clinoidectomy. **E.** Transpalpebral Approach. The intradural compartment communicated with the extradural compartment. The dissection allowed a wide surgical corridor from the peri-chiasmatic compartment to the cavernous sinus lateral wall. To increase visualization and internal carotid artery maneuverability, it is recommended to perform an anterior clinoidectomy followed by opening the proximal and distal dural ring. *Abbreviations*:FZS: frontozygomatic suture; TM: temporal muscle; ACP: anterior clinoid process; FL: frontal lobe; SOF, superior orbital fissure; TL: temporal lobe; ON: optic nerve; Ch: chiasma; cICA: internal carotid artery cavernous segment; III: oculomotor nerve; IV: trochlear nerve; V1: ophthalmic nerve; V2: maxillary nerve

The lateral extension of the approach allowed wide access to the temporalis muscle, which was detached from the superior temporal line until the pterion and greater wing of the sphenoid were exposed (Fig. 2A). A burr hole was drilled 5.0 mm superior from the pterion at the MacCarty keyhole to expose the anterior frontal dura mater and the periorbita. We opted for a large burr hole for safer use of the craniotome drill with a footplate attachment (Fig. 2B). Then, a craniotomy was executed in five cuts: (a) frontal; (b) orbital rim; (c) orbital roof; (d) greater wing of the sphenoid bone; and (e) zygomatic process. The final fracture of the orbital roof was performed with a KA chisel. This technique permitted a 1-piece frontoorbital approach. The margins of bone removal were chosen based on the landmarks: (a) the orbital rim osteotomy was extended to the supraorbital notch medially, while the lateral extension was represented by the area immediately below of the frontozygomatic suture; (b) the optic canal was used as medial and posterior extension for the orbital roof osteotomy, while the lateral extension of the osteotomy was represented by the area involving the greater wing of the sphenoid bone and zygomatic bone. Subsequently, the triangular anterior surface of the sphenoid was drilled, enabling visualization of the temporal and frontal dura mater. Comparing the TCA and TPA, a TPA took longer to perform, as the orbital rim, the greater wing of the sphenoid bone, and the zygomatic bone were included in the approach. After completion of the bone flap, the ACP was drilled using the extradural technique to achieve an enlarged view of the parasellar region (Fig. 2C and D). Lastly, the dura mater was opened in a C fashion based on the periorbita.

The dissection exposed the anterior cranial fossa through the subfrontal and pre-temporal corridor. First, we identified the ipsilateral optic nerve and internal carotid artery. Then, the ICA bifurcation, anterior cerebral artery (ACA), optic chiasm, anterior communicating artery (AcomA), the contralateral ICA, and the contralateral optic nerve were exposed (Fig. 2E). Finally, the Sylvian fissure was split, exposing the distal MCA branches. The limen insulae and the amygdala were identified.

## Results

### TCA dimensions

The average dimensions of our craniotomy for the TCA were 1.7 cm x 2.5 cm (Craniocaudal (CC) x lateral-lateral (LL)), ranging from 2.0 to 3.0 cm (LL) and 1.5 to 2.0 cm (CC) (Table 1). Both ACPs were identified and measured from the internal edge of the craniotomy in the TCA. The mean was 62 mm and 71.2 mm for the ipsilateral ACP and contralateral ACP, respectively. Analyzing the angle of attack and having the ipsilateral ACP as the landmark, the TCA allowed for an average CC angle of 14.9^°^.

**Table 1:** Craniotomy measurements, angle of attack, ipsilateral, and contralateral anterior clinoid process measurements obtained with each specimen and approach

Approach	Specimen	Lateral-Lateral	Cranial-Caudal	Ipsilateral ACP	Contralateral ACP	Angle of Attack (°)
**Transciliary**	1	2.5 cm	1.5 cm	61 mm	72 mm	14.4
	2	3.0 cm	2.0 cm	62 mm	71 mm	15.3
	3	2.0 cm	1.5 cm	63 mm	71 mm	15.7
	4	2.5 cm	1.5 cm	61 mm	72 mm	14.2
	5	2.5 cm	2.0 cm	63 mm	70 mm	15
	Average	2.5 cm	1.7 cm	62 mm	71.2 mm	14.9
**Transpalpebral**	1	3.0 cm	2.0 cm	48 mm	62 mm	8.1
	2	3.0 cm	2.5 cm	47 mm	63 mm	9
	3	3.0 cm	1.5 cm	47 mm	64 mm	7.9
	4	3.0 cm	2.5 cm	48 mm	62 mm	8.5
	5	2.5 cm	2.0 cm	49 mm	63 mm	8
	Average	2.9 cm	2.1 cm	47.8 mm	62.8 mm	8.3

**Abbreviations.** APC: Anterior Clinoid Process

### TPA dimensions

The mean craniotomy size was 2.1 cm x 2.9 cm (CC x LL) for the TPA, ranging from 2.5 to 3.0 cm (LL) and 1.5 to 2.5 cm (CC) **(**Table 1**)**. Using the same principle applied to the TCA, the mean for TPA was 47.8 mm and 62.8 mm for the ipsilateral ACP and contralateral ACP, respectively. Using the ipsilateral ACP as a landmark, the TPA allowed for a cranial-caudal angle average of 8.3^°^. It is essential to point out that the sample sizes were too small to perform statistical analysis on the cadaver measurements.

### Analysis of the extension

**Fig. 3:**
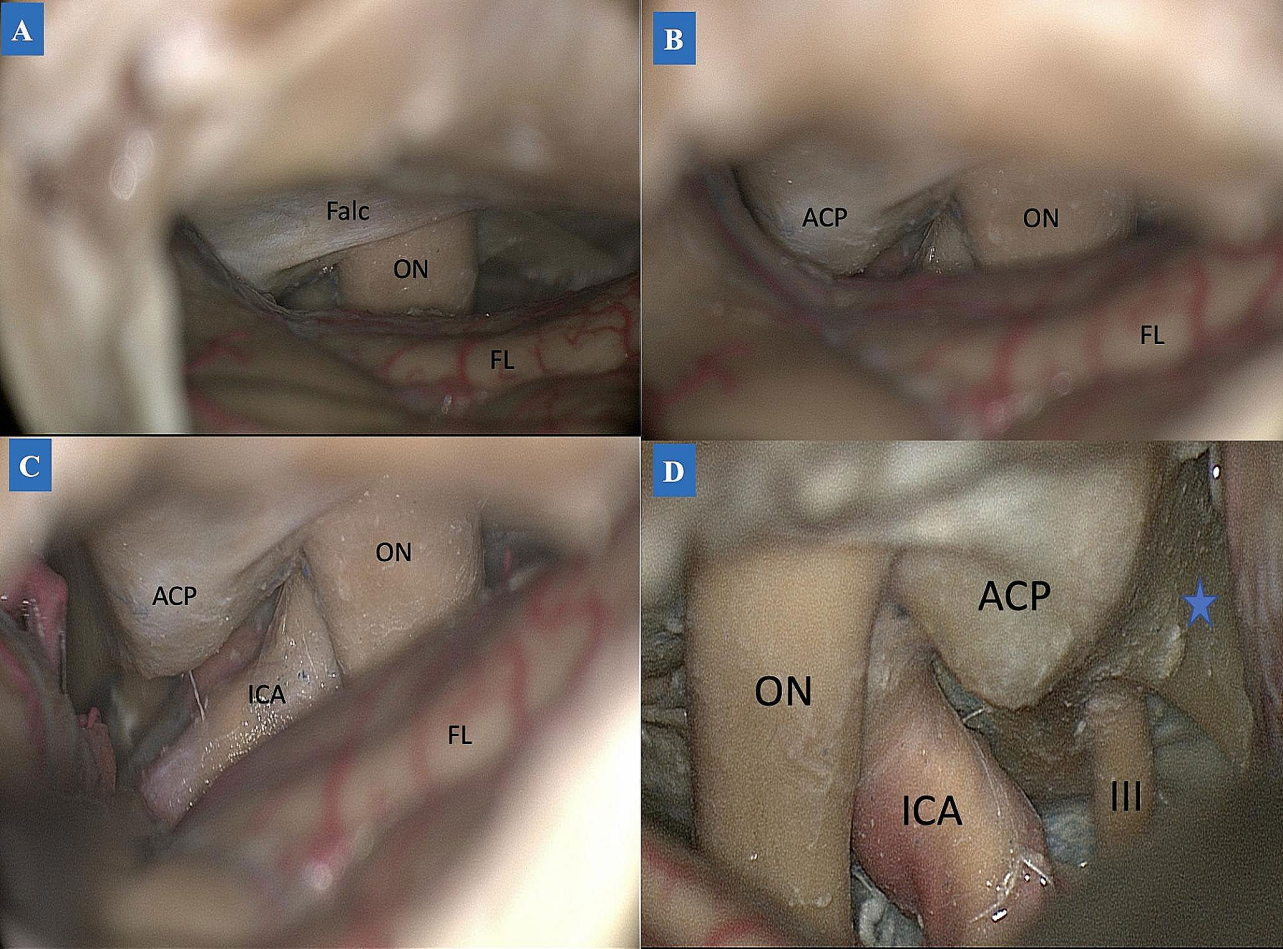
TCA subfrontal dissection. **(A-C: left side) A**: During the dissection, the ipsilateral optic nerve was first accessed on the anterior skull base, with the falciform ligament covering the optic canal roof. **B**: Following the dissection laterally, the anterior clinoid process was exposed. **C**: This stage was characterized by internal carotid artery exposure. **D (right side).** Advanced dissection showing the lateral wall of the cavernous sinus (blue star). *Abbreviations*: ACP: anterior clinoid process; Falc: falciform ligament; FL: frontal lobe; ICA: internal carotid artery; ON: optic nerve; III: oculomotor nerve

The TCA allowed access to half of the sphenoid bone contralaterally and to the medial two-thirds of the sphenoid bone ipsilaterally **(**Fig. 3**)**, while the TPA guaranteed access to one-half of the contralateral sphenoid wing. At the same time, the ipsilateral limit included all aspects of the sphenoid wing. Accessible structures in each approach demonstrated in Table 2. A partial anterior clinoidectomy was performed through an intradural corridor in the TCA, while the same procedure was carried out through an extradural corridor in the TPA, obtaining a complete anterior clinoidectomy. Comparatively, the anterior clinoidectomy was performed more accurately in the latter. The TPA allowed larger access to the temporal region, including the temporal pole, the Sylvian fissure up to the M4 segment of the MCA, the limen insulae, and the amygdala. Besides, this approach allowed for the peeling of the middle fossa, followed by a transcavernous dissection into the direction of the interpeduncular fossa.

**Table 2:** Comparative Analysis of Transciliary Approach (TCA) and Transpalpebral Approach (TPA)

	TCA	TPA
Average craniotomy size (CC x LL)	1.7 cm x 2.5 cm.	2.1 cm x 2.9 cm.
Mean CC angle	14.9◦ (14.2° − 15.3°).	8.3◦ (7.9° − 9.0°).
Identified anatomic landmarks	Ipsilateral ICA, ICA bifurcation, M1 segment of the MCA, ipsilateral and contralateral ACA (A1 segment), AComA, ipsilateral and contralateral optic nerve, and optic chiasm.	Ipsilateral ICA, ICA bifurcation, M1 segment of the MCA, ipsilateral and contralateral ACA (A1 segment), AComA, ipsilateral and contralateral optic nerve, and optic chiasm. Temporal pole, Sylvian fissure (M1-M4 of the MCA), the limen insulae, and the amygdala.
Sphenoid bone access	Half contralateral, medial two-thirds ipsilateral SPW.	Half contralateral, complete ipsilateral SPW.
Surgical corridor advantages	Better visualization of midline anterior cranial fossa.	It improved lateral-medial visualization of the anterior fossa after sphenoid bone and orbital roof drilling. Access to the middle fossa and temporal region.
Postoperative complications	Risk of frontalis palsy. Scar and cosmetic issues.	Compartment-like syndrome. Infectious blepharitis. Ocular manipulation.
Key-considerations	- Faster execution. - Better cranial-caudal angle of attack. - Potential cosmetic concerns.	- Optimal for pathologies involving anterior and middle fossa. - Better caudal-cranial angle of attack. - Potential ocular concerns. - More time consuming due to orbital osteotomy and wider drilling of the SPW

**Abbreviations.** TCA: Transciliary approach, TPA: Transpalpebral approach, ACA: anterior cerebral artery, LL: Lateral-Lateral, AComA: anterior communicating artery, A1: first segment of the anterior cerebral artery, ICA: internal carotid artery, MCA: middle cerebral artery, M1: first segment of the middle cerebral artery, M4: fourth segment of the middle cerebral artery, SPW: sphenoid wing

## Discussion

**Fig. 4:**
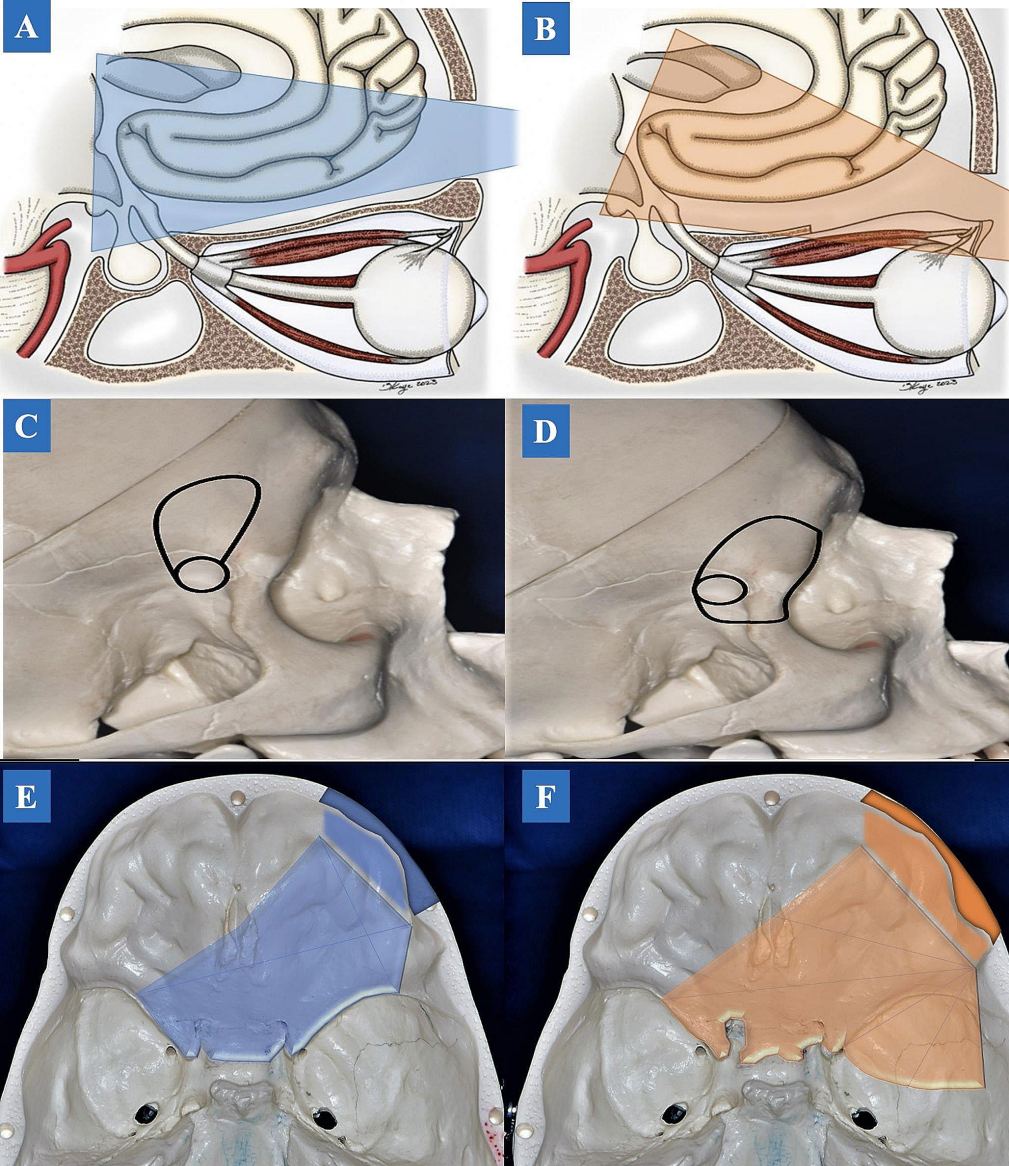
Illustration drawing. The angle of attack between the TCA and TPA. **A**. TCA allows a better cranial-caudal angle of attack. **B**. In TPA, the angle of attack is directed from a caudal-cranial perspective. The advantage is that after drilling the orbital rim and orbital roof. An option is to use a Layla retractor to dislocate the periorbita from the surgical field. **C**: Dry skull drawing of TCA. Traditionally, we tend to preserve the orbital rim for that approach, although it may be included if needed. **D**: Dry skull drawing of TPA (Orbitotomy is part of the approach for a more lateral extension). The TPA has the advantage of a lateral extension. **E**: Dry Skull Base Drawing of Right-side TCA. It is possible to access most of the anterior cranial fossa up to the midpoint of the contralateral sphenoid ridge and the surroundings of the crista galli. Although it may be possible, the temporal lobe access is limited by the approach anterior perspective. **F**: Dry Skull Base Drawing of Right-side TPA. Roughly, most of the anterior cranial fossa landmarks are achieved by the TPA like the TCA; however, there is a slight advantage to accessing the more anterior portion of the crista galli, as the TPA allows a better vision from a lateral-medial perspective. Moreover, the benefits are clearly represented by accessing the middle cranial fossa and temporal lobe due to approach lateral extension. *Abbreviations*: TCA: transciliary approach; TPA: transpalpebral approach

While both approaches aim to access the anterior cranial fossa, each has its precise nuances. The TCA performs like a subfrontal approach with the advantage of a better cranial-caudal angle of attack. Whereas the TPA, through a transorbital corridor, provides a better caudal-cranial angle of attack, favoring the resection of lesions located around the third ventricle, and extending to the temporal lobe **(**Fig. 4A and B**).** The advantages of both these minimally invasive approaches include a shorter intra-operative time, decreased brain tissue manipulation, excellent visualization and maneuverability, and great cosmetic outcomes [11, 12].

### Cosmetic outcomes

Although we had performed a transciliary incision, it is possible there are variations, such as a supra or infraciliary incision. The temporal branch of the facial nerve is located approximately 2.54 cm lateral and 2.85 cm superior to the lateral canthus on the eye [13]. Thus, there may be a higher risk of postoperative frontalis muscle palsy with the TCA if a lateral extension is required beyond the lateral edge of the eyebrow. However, a large case series on 418 patients who underwent a TCA showed that more than 75% of patients reported very pleasant satisfaction [4]. Regarding worse TCA cosmesis, one related aspect may be the infection process, which results in disfiguring results. If this process is avoided, the results will be satisfactory, as demonstrated by Avery et al., when there was only one case of wound infection among 173 TCA cases [10].

In contrast, the TPA avoids postoperative frontalis muscle palsy as the incision is opposite from the nerve trajectory. Elnokaly et al. showed excellent cosmetic outcomes based on imperceptible incision line, scarring, or temporalis atrophy at follow-up, in 43 of 44 patients who underwent the transpalpebral approach [14]. Mandel et al. also reported excellent cosmetic results in a prospective study of 25 patients who underwent elective aneurysm clipping via TPA. Among patients, nobody noted the prominence of the cranial fixation hardware on the frontozygomatic process [15]. While the TCA can provide good cosmetic results as shown by multiple studies [16–18], the incision is always visible and apparent. On the other hand, the TPA approach offers cosmetic results that are not noticeable after a couple of months of the surgery as the incision is hidden inside the eyelid crease.

### Ultrasound surgery and orbital reconstruction

Two characteristics that must be pointed out are the application of piezoelectric or ultrasound surgery and orbital reconstruction after TPA. One of the benefits of applying ultrasonic vibrations to perform osteotomies in TPA is to decrease damage to surrounding structures, such as periorbita, optic nerve, or lacrimal gland; conversely, it is more time-consuming than traditional bone drill [19]. In relation to the necessity of wall reconstruction to prevent enophthalmos, some authors argue that it is unnecessary [20, 21]. However, other skull base surgeons affirm that this approach prevents pulsating enophthalmos, orbital pain, and restrictive ptosis [22]. When discussing reconstruction materials, it’s essential to mention autogenous bone grafts, titanium meshes, or polymethyl methacrylate (PMMA). Additionally, certain techniques have proven beneficial in supplanting complex orbital volume. For example, computer-assisted design (CAD) and computer-assisted manufacture (CAM) can be utilized to fabricate customized implants [22].

### Size of craniotomy and angle of attack

For the TCA, our findings confirmed previous published results with an average supraorbital craniotomy area of 4.25 cm^2^ [2, 10, 23]. It is important to note, that in specimens where the craniotomy for the TCA was smaller than 3.75 cm^2^ (2.5 cm LL x 1.5 cm CC), instruments could still be manipulated comfortably. However, it is crucial that the surgeon uses long, bayoneted instruments to navigate the narrow and deep surgical corridors (Fig. 4C and D).

In the clinical setting, every inch of space is important and must be optimized. Therefore, TPA may be advantageous as its craniotomy area was slightly larger than the TCA. This will optimize the surgical field and enhance the microscopic visualization. It also makes it feasible for endoscopic approaches, as the endoscope will not collide with the other surgical instruments. With the ipsilateral anterior clinoid process serving as a reference point, the TCA facilitated an average angle of 14.9^°^ for the CC angle, while the TPA yielded an angle of 8.3^°^(Table 1). These results were similar to that of Rychen et al., which were 15.7^°^ and 6.5^°^, respectively^2^. Thus, in the CC angle, the TCA allows for a wider view and better angle of attack, as reported by the illustrative case 1 (Video 1). Additionally, this working angle can be further improved by the removal of the orbital rim [23].

During cadaveric dissections, the TPA was performed via a standard orbital-frontal craniotomy, encompassing the orbital rim and the lateral aspect of the orbital wall, extending inferiorly to the zygomatic frontal suture. These aspects were deemed crucial for a larger craniotomy compared to a TCA. While it’s feasible to execute a small craniotomy confined to the orbital rim without engaging the lateral portion of the orbital bone, this approach may result in a narrow surgical corridor, thereby restricting access to the middle fossa.

The TPA creates a tangential view of the anterior skull base. A smaller angle of attack offered by the TPA may be a drawback when we consider surgeries involving the anterior skull base. On the other hand, a greater angle of attack, as the TCA provides, allows for a more perpendicular view of the anterior skull base. This leads to better control of the lesion and identification of important neuroanatomic landmarks. Although, we have not coupled with an orbital osteotomy during dissections, this maneuver in the TCA provides an even better CC angle of attack for the management of lesions located around the third ventricle such as retrochiasmatic craniopharyngiomas or bulky tuberculum sellae meningiomas.

### Anterior and middle cranial fossa

**Fig. 5:**
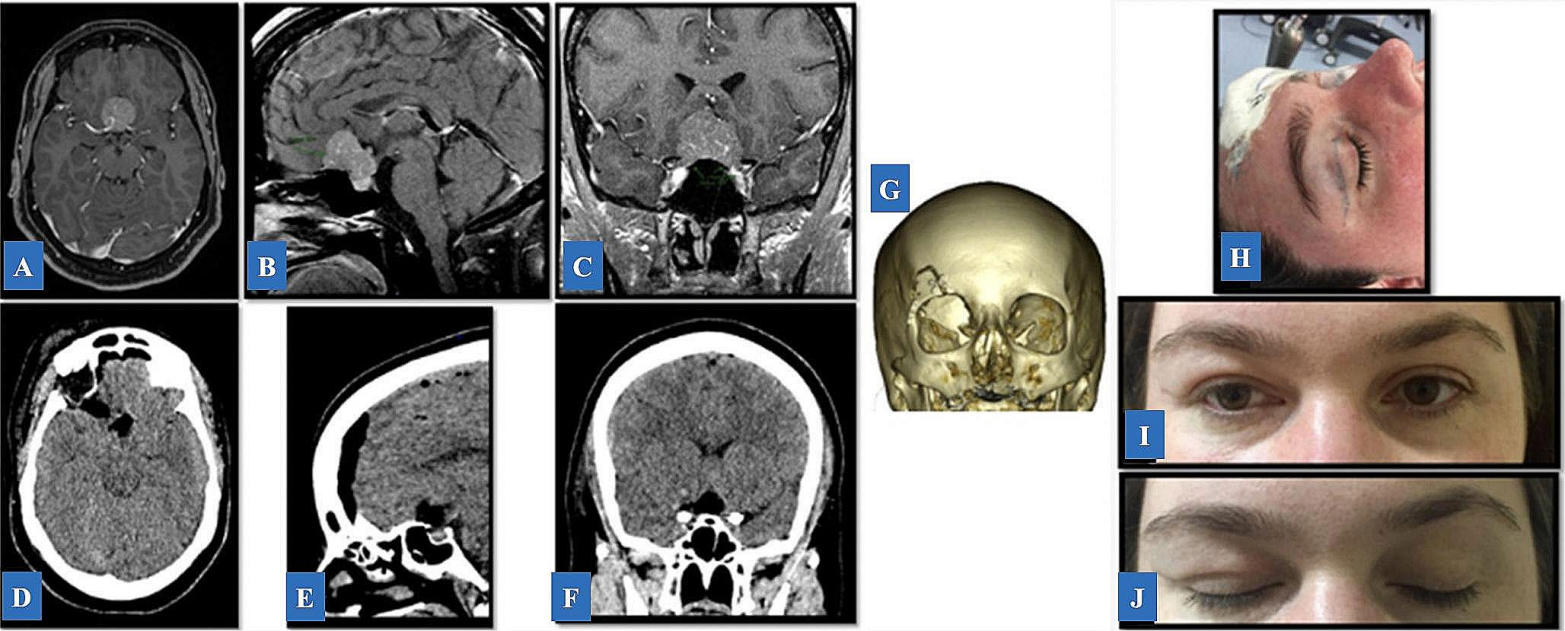
Illustrative Case 2. **A-C**: Pre-operative T1-weighted post-contrast magnetic resonance imaging demonstrating an extra-axial, enhanced lesion in the tuberculum extending to the planum sphenoidale. **D-F**: Post-operative brain computed CT scan demonstrating gross total resection. **G**: A 3D-reconstructed CT scan demonstrating the small size of the craniotomy and an adequate reconstruction using plates and self-drilling screws. **H**: A 3.5 incision following an eyelid crease on the right side was marked with the head in a neutral position with a mild cervical extension. **I-J**: Post-operative view showing an aesthetical outcome

Based on our results, it was possible to access most of the landmarks on the anterior skull base with either a TCA or a TPA **(**Fig. 4E and F**)**. For both approaches, the contralateral edge was represented by the midpoint of the sphenoid ridge [24]. It is important to emphasize the effect of cerebrospinal fluid (CSF) drainage and its effect on brain tissue relaxation and enhanced exposure. This cannot be appropriately evaluated with fixed anatomical specimens and plays an important role in selecting the surgical approach. For this we present an illustrative case, using the TPA for a tuberculum sellae meningioma **(**Fig. 5A and J**)**. Furthermore, the midpoint of the crista galli and the ipsilateral olfactory groove were identified in both approaches. These two structures were better identified through a TCA because of the favorable craniocaudal angle. However, the same structures could be accessed with a TPA after drilling the orbital roof and pulling away the periorbital fascia. These results differed from previous studies where the posterior aspect of the crista galli and the ipsilateral olfactory groove were identified in the TCA without drilling the orbital roof [24]. In the clinical setting, our findings reinforce the applicability of minimally invasive surgery for most anterior skull base lesions. However, due to the inherent blind spots as discussed above, we would recommend the addition of other visualization methods like endoscopy to treat lesions of the olfactory groove. There is also an alternative method which is characterized by an endoscopic endonasal approach combined with a TCA since the combination of these two approaches could allow for gross total resection of the lesion as there is an overlap on the midorbital line between the approaches [25]. Additionally, all cadaveric specimens were classified as Keros type 1. In other words, the depth of the olfactory fossa was between 1 and 3 mm. Indeed, this condition created a favorable identification and dissection of the olfactory fossa [26].

### Endoscopic transorbital approach

In recent years, novel strategies have emerged to enhance minimally invasive skull base surgery. Transorbital neuroendoscopic surgery (TONES) has garnered increasing attention among skull base surgeons, particularly for treating Spheno-Orbital Tumors. While our anatomical dissections have not specifically explored this approach, it shares common principles with transpalpebral approache, particularly in addressing middle fossa pathologies such as spheno-orbital lesions [27]. In essence, the endoscopic transorbital approach utilizes the orbit as a surgical corridor, accessed through a superior eyelid incision, to address various pathologies ranging from cerebrospinal leaks to skull base tumors [28]. When considering this approach to the middle fossa, several key landmarks come into play: superiorly, the lesser sphenoid wing; inferiorly, the lateral pterygoid muscle; laterally, the foramen spinosum, housing the middle meningeal artery; and medially, the Gasserian ganglion and the lateral edge of the foramen rotundum [29]. Taking into account the findings of Guizzardi et al., this approach demonstrates a tendency to achieve a middle fossa floor osteotomy safely in up to 63% of the middle fossa bone volume. Consequently, its extension permits access to the clivus and the posterior cranial fossa in specific instances [29]. Similar to endoscopic endonasal transsphenoidal surgery, the endoscopic transorbital approach exhibits a learning curve that can be categorized into five stages: Stage 1 involves addressing extraconal lesions, extradural lesions, and posttraumatic CSF leaks. Stage 2 entails managing intradural tumors, such as spheno-orbital meningiomas. Stage 3 is focused on treating intraconal lesions and tumors surrounding Meckel’s cave. Stage 4 involves handling lesions involving the anterior clinoid process and cavernous sinus. Finally, Stage 5 addresses tumors affecting the petrous apex or pathologies within the insula or Sylvian fissure [30]. While the endoscopic transorbital approach shows promise in ameliorating proptosis, visual deficits, and diplopia in patients with spheno-orbital tumors, as reported by Zoli et al., postoperative ocular complications pose significant concerns. These complications include extrinsic ocular muscle deficits, enophthalmos, and macular hemorrhage [27]. To mitigate these risks, various strategies have been proposed, such as clinical assessment of pupil size and shape, intermittent release of ocular retraction, and intraoperative tonometry [29].

### Anterior clinoidectomy

An anterior clinoidectomy is sometimes imperative for managing complex tumors or aneurysm clipping. Despite the execution of anterior clinoidectomy in both approaches, there were distinctions in the techniques employed. In the TCA, the anterior clinoidectomy was conducted by an intradural technique, and given the limited surgical field, this represented a challenge. Thus, an endoscopy-assisted technique may be useful in increasing the surgical field visualization and enhancing the safety of the procedure. By contrast, the TPA allowed for an anterior clinoidectomy through an extradural technique. This created an enlarged view of the ACP and increased the instrument’s maneuverability due to an oblique perspective of the surgical field. Overall, a TPA might be the better choice if an anterior clinoidectomy is needed.

### Aneurysms

The TPA demonstrated a favorable corridor to access the middle fossa and allowed for complete Sylvian fissure dissection, exposing the ICA and MCA branches. We also performed extradural anterior clinoidectomy through a TPA, allowing for better visualization of the parasellar region, besides the opticocarotid and carotid-oculomotor triangles as well as adequate mobilization of the ICA after cutting the distal and proximal ring. In congruence with our findings, Mandel et al. demonstrated that the TPA provided direct visualization of the MCA bifurcation when this approach was performed in 25 patients with unruptured MCA aneurysms [15]. Additionally, a systematic review showed the applicability of TCA for aneurysms on 1413 patients (34.7%) and 1142 patients (28.0%) with AcomA, and MCA aneurysms, respectively [31]. Although either the TCA or TPA may be used for clipping aneurysms located in the MCA, we found that the TPA provided a better exposure of the MCA and distal branches. Therefore, we recommend the TPA for clipping non-giant MCA aneurysms.

### Frontal lesions

The same principle may be applied to lesions at the frontal pole including metastases or gliomas. We had difficulty reaching the superior-frontal edge of the brain with either approach. To overcome this obstacle, we used endoscopic dissection (0^◦^ and 30^◦^), correcting the blind spot and improving the view of the surgical field. Nevertheless, even though the endoscope is recognized as an essential tool in skull base surgery or the keyhole craniotomy approach, Robinow et al. showed that its use for tumor cases did not represent a better outcome [31]. Additionally, the endoscope-assisted vascular category in the same meta-analysis showed a marginally lower mean technical success than the no-endoscope-assisted vascular category which may be related to the endoscope-assisted neurosurgery learning curve [31].

### Temporal lesions

**Fig. 6:**
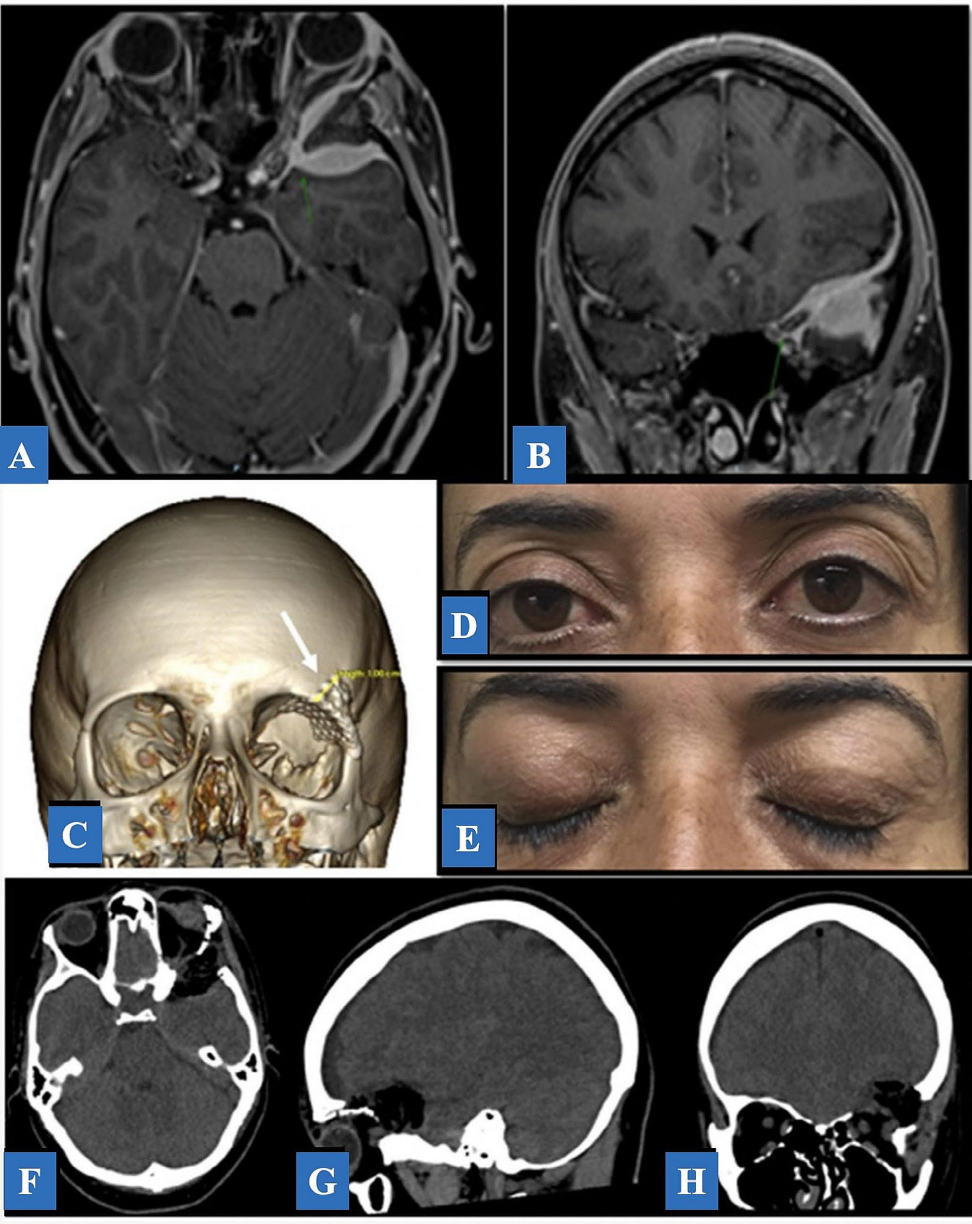
Illustrative Case 3. **A**, **B**: Axial and coronal T1-weighted with contrast MRI post-contrast demonstrating an extra-axial, enhancing, spheno-orbital left-sided lesion. **C**: A 3D-CT reconstruction demonstration of a satisfactory cranioplasty using titanium mesh (white arrow). Although there were not related complications, the use of bare titanium mesh may cause dural damage as a result from dural pulsations against the sharp edges over time. Currently, we do not use it in clinical setting. **D**, **E**: Post-operative picture showing an aesthetically acceptable result, the proptosis was completely resolved, and no enophthalmos was reported. **F-H**: Post-operative CT brain demonstrating gross total resection of the lesion

During the dissections, it was possible to access the Sylvian fissure and dissect it using the TPA, exposing it from the ICA bifurcation to MCA M4 branches. The advantage was access from an anterior perspective. This advantage related to the TPA is useful when lesions are in the temporal pole, as shown in illustrative case 3 (Fig. 6A and H). This corridor exposed the anterior half of the temporal lobe, including the anterior portion of the uncus. In a clinical setting, surgeons may perform this approach for temporal lobectomies or selective amygdalohippocampectomy. However, an endoscopic-assisted surgery might be required due to insufficient angle of vision following the microscopic amygdala resection alone [32]. There is also a significant limitation to the insular region, which makes pathology in this region very difficult to treat through either the TCA or TPA. Similarly to Raza et al. study, we suggest a TCA for the situations where there is no necessity for an orbital osteotomy and a TPA for the situations where one intends to perform an orbital osteotomy [33]. Analyzing both approaches, the TPA allowed for more ergonomic performance, especially when accessing the anterolateral corridor, like the views and working angle of a traditional modified orbital-zygomatic approach. However, in the TPA it is crucial to remove the orbital rim and retract the periorbita, as it maximizes the surgical corridor.

### Risks of each approach

Although minimally invasive surgery represents an advance in skull base surgery, each approach carries risks and limitations. Common risks for both techniques are inadequate exposure, limited maneuverability, and rarely poor cosmesis.

#### Transpalpebral approach

When assessing potential complications, it’s important to recognize that they are primarily associated with orbital-frontal craniotomy and orbital roof drilling. In essence, complications arising from the endoscopic transorbital approach should be considered collectively. The transorbital approach carries the risk of damaging the elevating muscle of the upper eyelid during palpebral incision or the lacrimal gland during periorbita detachment. Particularly in older patients, there is an increased risk of periorbital region lesions with fat extrusion into the surgical field, as well as the potential for orbital compartment syndrome if the orbital rim reconstruction is inadequate. In a systematic review conducted by Zoli et al., complications associated with the endoscopic transorbital approach for spheno-orbital tumors encompassed various issues such as scar problems or infections, persistent or significant palpebral edema, cerebrospinal fluid leaks, macular hemorrhage, chronic subdural hematoma, trigeminal hypoesthesia, diplopia or extrinsic ocular muscle palsy, and visual acuity deficits, with prevalence rates ranging from 0.8 to 4.7% [27].

#### Transciliary approach

There are three variations of incision types: trans, supra, or infraciliary; however, the transciliary incision may yield better cosmetic results [34]. Another concern involves the risk of damaging the supratrochlear or supraorbital nerves; one preventive measure is to perform the incision laterally. Additionally, the TCA poses an elevated risk of transient or permanent frontalis palsy. Given that the TCA is confined to the frontal bone, there is a potential risk of frontal sinus opening during frontal craniotomy, predisposing to meningitis or CSF leak. To mitigate this risk, an alternative approach involves utilizing the neuronavigation system to avoid the frontal sinus or employing a fat graft to obstruct it if opened. Furthermore, when the TCA is employed to address lesions involving the internal carotid artery, optic nerve, or anterior clinoid process, inadequate proximal control heightens the risk of damaging these structures. In a systematic review conducted by Zumofen et al., involving 2783 patients undergoing a TCA, approach-related complications ranged from 1 to 4.3%. These complications encompassed cerebrospinal fluid collection or leak, permanent or temporary supraorbital hypesthesia, permanent or temporary facial nerve palsy, and wound healing disturbances. Furthermore, complete tumor resection was achieved in 90% of cases [18]. Another systematic review by Robinow et al. reported major complications in 46.5% of cases, minor complications in 53.5% of cases, and a mean treatment-related mortality of 1.26% [31].

### General considerations

We do not advocate for minimally invasive surgery for all cases. As affirmed by previous studies, care must be taken to not try and advance an approach beyond its utility or capability [24, 25]. We suggest initially gaining experience through the so-called classic approaches (e.g., pterional approach) and progressively migrating to minimally invasive techniques once the comfort level and ability have grown. As Andaluz et al. have recommended, the best initial cases may be represented by unruptured aneurysms of the anterior circulation with favorable angiographic features and anterior skull base tumors involving the posterior planum or tuberculum [5]. We also recommend the use of advanced tools such as neuronavigation and Doppler to increase the safety and efficiency of these surgeries.

In general, a TCA may be recommended for pathologies situated in the anterior cranial fossa and for patients at higher surgical risk, owing to its swifter surgical procedure. Conversely, a TPA can address lesions not only in the anterior cranial fossa but also those originating from the middle cranial fossa. However, it is a more time-consuming procedure and demands a deeper understanding of neuroanatomy, including its anatomical landmarks. Furthermore, a TPA may offer superior management of multicompartmental lesions [35].

### Limitations

It is essential to point out that the sample sizes were too small to perform statistical analysis on the cadaver measurements, and there were correlated limitations, resulting in a smaller range of anatomic variations to explore. Additionally, the specimens did not represent models of skull base tumors or vascular malformations. This contributed to standard dissections, not considering the obstacles and difficulties related to these pathologies. Besides, as with any anatomic cadaveric dissection, the specimens do not represent in-vivo tissue compliance or surgical procedural morbidity like in a clinical setting. This is especially important, as an essential step during both approaches is brain relaxation obtained by CSF drainage which is not feasible in cadaveric specimens. Indeed, the extension of each approach may be underestimated in a cadaveric specimen, as “brain sag” may be present in a clinical setting. Lastly, the points discussed here disclose an overview and not a strict pattern for each clinical case.

## Conclusion

Considering the TCA, the advantage is the lesser operative time, and better visualization of the anterior cranial fossa due to a sizeable cranial-caudal angle of attack, with a shorter learning curve. In contrast, the TPA represents the best option for pathologies localized in the middle fossa, including those in the anterior temporal lobe. It also provides a favorable visualization from the lateral to the medial aspect of the anterior fossa, after drilling the lesser and greater wing of the sphenoid bone. This approach also improves the caudal-cranial angle of attack. However, the learning curve is longer as eyelid anatomy is not familiar territory for most neurosurgeons. Our study demonstrates that these approaches are not variants of one another but instead different approaches that provide distinct advantages based on the surgery type. When appropriately chosen, both approaches may provide a comfortable and safe surgical field, resulting in less traumatic and painful surgery, and shorter hospital stays.

## Electronic supplementary material

Below is the link to the electronic supplementary material.

Supplementary Material 1: Video Legend Illustrative case 1: We present the case of a 50-year-old woman, with a chief complaint of a 5-year history of frontal headache. She had an unremarkable neurological exam, and a brain MRI demonstrated a homogenously enhancing anterior skull base mass, presumably a meningioma originating from the orbital roof. The patient underwent microsurgical resection via a keyhole eyebrow supraorbital craniotomy. After the craniotomy, the dura was opened in a C-shaped manner and reflected anteriorly. CSF was drained to aid brain relaxation, and the tumor was de-vascularized from its base. After adequate devascularization, the tumor was biopsied and sent for histo-pathology assessment. The bony tubercle (presumably the origin of the tumor) was then drilled. The tumor was further debulked using an ultrasonic aspirator. The tumor was then mobilized, separated, and removed. Final bits of the tumor were taken, and hemostasis was obtained. The remaining tubercle was drilled flush to the orbital roof, after which the resection site was copiously irrigated, hemostasis was verified, and closure was performed. The patient’s pathology was consistent with a WHO grade 1 meningioma. Post-operative CT showed complete resection of the lesion. She did well following surgery and was discharged on post-operative day one. Subsequent follow up did not reveal any signs of tumor recurrence.

## Data Availability

No datasets were generated or analysed during the current study.
